# THE IMPACT OF A SERVICE-LEARNING COURSE ON UNDERGRADUATE STUDENT ATTITUDES TOWARD OLDER ADULTS

**DOI:** 10.1093/geroni/igad104.0369

**Published:** 2023-12-21

**Authors:** Britteny Howell

**Affiliations:** University of Alaska Anchorage, Anchorage, Alaska, United States

## Abstract

Undergraduate students often have little contact with older adults, which may result in a lack of interest regarding career options in gerontology or geriatric practice. To combat this problem, the University of Alaska Anchorage’s 300-level Public Health for an Aging Society course introduces students enrolled in the Health Sciences Bachelor’s program to a variety of gerontological concepts. This service-learning course engages public health and pre-clinical students in the community, providing valuable experience working with local senior agencies to provide a product or service. Students work in interdisciplinary teams and have a variety of community-based projects to select from as part of their coursework. Using the Geriatric Attitudes Scale at the start and end of the semester, this presentation reports on improved student perceptions of older adults as a result of this course. With a sample of 22 undergraduates at the beginning of the semester, scores were relatively high in agreement with positive statements about aging (i.e., “it is society’s responsibility to provide care for the elderly”). However, they also report agreement with less positive statements about aging, such as “taking a medical history from elder patients is frequently an ordeal” and “as people grow older, they become less organized and more confused.” At the conclusion of the 15-week course, students report lower agreement with these negative statements about aging as a result of their learning and hands-on experience.

